# Extracorporeal Membrane Oxygenation in Refractory Cardiac Arrest: Current Evidence, Clinical Pathways and Future Directions

**DOI:** 10.3390/life16050857

**Published:** 2026-05-21

**Authors:** Debora Emanuela Torre, Domenico Mangino, Carmelo Pirri

**Affiliations:** 1 Department of Cardiac Anesthesia and Intensive Care Unit, Cardiac Surgery, Ospedale Dell’Angelo, 30174 Venice, Italy; deboraemanuela.torre@aulss3.veneto.it; 2 Cardiac Surgery Department, Ospedale Dell’Angelo, 30174 Venice, Italy; domenico.mangino@aulss3.veneto.it; 3 Department of Neurosciences, Institute of Human Anatomy, University of Padova, 35121 Padua, Italy

**Keywords:** extracorporeal cardiopulmonary resuscitation, ECPR, cardiac arrest, veno-arterial ECMO, refractory cardiac arrest, resuscitation system, post-resuscitation care

## Abstract

**Background**: Extracorporeal cardiopulmonary resuscitation (ECPR) has emerged as a promising strategy for refractory cardiac arrest, enabling the restoration of systemic perfusion when conventional resuscitation fails. However, uncertainties remain regarding patient selection, timing and implementation. **Methods**: A narrative review of experimental data, clinical studies, randomized trials and international recommendations was performed. Particular emphasis was placed on the interplay between physiological mechanisms, real-world organizational models and decision-making processes. **Results**: ECPR can restore effective circulation, preserve end organ perfusion and serve as a bridge to definitive etiologic treatment, with the potential to improve survival and neurological outcomes in highly selected patients. However, its effectiveness is strongly dependent on rapid deployment, structured systems of care and multidisciplinary coordination. Significant challenges remain, including in relation to the heterogeneity of protocols, high resource utilization, complications with extracorporeal support and the complexity of post-resuscitation management. Furthermore, ECPR fundamentally alters traditional resuscitation paradigms, introducing ethical dilemmas related to patient selection, prognostication and the allocation of limited resources. **Conclusions**: ECPR represents a transition from procedure-based resuscitation to system-based extracorporeal support. Its clinical benefit is contingent upon timely implementation within optimized organizational frameworks and integration with definitive treatment pathways. Future research should focus on refining selection criteria, standardizing care pathways and addressing ethical sustainability challenges to ensure appropriate and effective use of this evolving technology.

## 1. Introduction

Cardiac arrest remains one of the leading causes of death worldwide and continues to represent a major public health challenge despite decades of advances in resuscitation science. Survival rates after out-of-hospital cardiac arrest (OHCA) remain disappointingly low and even in-hospital cardiac arrest (IHCA) is frequently associated with poor neurological outcomes [1]. Conventional cardiopulmonary resuscitation (CPR), although foundational, restores only a fraction of normal cardiac output, resulting in persistent systemic hypoperfusion, metabolic acidosis and progressive multiorgan injury [2]. Extracorporeal cardiopulmonary resuscitation (ECPR), defined as the rapid deployment of veno-arterial extracorporeal membrane oxygenation (V-A ECMO) during ongoing CPR, has emerged as a transformative strategy for patients with refractory cardiac arrest. By restoring circulatory and respiratory support, ECPR creates a therapeutic window during which reversible causes, such as acute coronary occlusion, pulmonary embolism, refractory arrhythmias or profound myocardial stunning, can be identified and treated [3,4]. The physiological rationale for ECPR lies in its capacity to replace the severely compromised hemodynamic output generated by chest compressions. During conventional CPR, cardiac output typically reaches only 20–30% of baseline, which is often insufficient to prevent irreversible ischemic injury [2,3,5]. V-A ECMO can rapidly re-establish near-normal systemic blood flow, restore oxygen delivery and mitigate the inflammatory and ischemia–reperfusion cascade that follows prolonged low-flow states [3,5,6]. This paradigm reframes cardiac arrest from an inevitably terminal event to a potentially reversible state of profound but time-dependent circulatory failure. Observational data and registry analyses have suggested an association between ECPR and improved survival with favorable neurological outcomes in selected patients [7,8,9,10]. However, these findings should be interpreted with caution, as they are inherently subject to selection bias, survival bias and confounding related to system performance and institutional expertise [11,12,13]. These findings suggest that ECPR outcomes are closely influenced by organizational efficiency, rapid deployment and multidisciplinary coordination. In this context, ECPR can be conceptualized as a time-sensitive circulatory replacement therapy constrained by ischemic tolerance thresholds, in which the interval from cardiac arrest to effective perfusion represents the dominant determinant of outcome. High-volume ECMO centers and integrated regional networks appear to achieve superior outcomes, suggesting that organizational structure is as important as technological capability [14,15,16]. Despite its growing adoption, major uncertainties remain. Optimal patient selection criteria, the acceptable duration of low-flow before initiation, the management of complications such as left ventricular distension and differential hypoxia, long-term neurological prognostication and the ethical implications of resource-intensive resuscitation in marginal candidates are still incompletely defined. Furthermore, the expansion of mobile ECPR teams and prehospital ECMO programs raises new logistical and equity considerations. ECPR represents a paradigm shift from conventional CPR toward an integrated extracorporeal life support strategy embedded within advanced cardiac care system. However, its effectiveness remains highly dependent on patient selection, timing and organizational expertise. In this narrative review, we critically examine the physiological rationale, clinical evidence, technical aspects and organizational challenges of ECPR in refractory cardiac arrest.

## 2. Materials and Methods

This study was conducted as a narrative review aimed at integrating current evidence on extracorporeal cardiopulmonary resuscitation (ECPR) in refractory cardiac arrest, with a specific focus on the interplay between pathophysiology, clinical outcomes and system-level implementation. A literature search was performed using PubMed/MEDLINE, Scopus and Embase, covering the period from January 2010 to February 2026, with selected seminal pre-2010 publications included when relevant to foundational pathophysiology, the historical evidence or the conceptual background. The search strategy combined the following keywords and Medical Subject Headings (MeSH) “extracorporeal cardiopulmonary resuscitation”, “ECPR”, “veno-arterial ECMO”, “cardiac arrest” and “refractory cardiac arrest”. Additional relevant studies were identified through the manual screening of reference lists from key articles and recent reviews. The inclusion criteria comprised randomized trials, large observational cohort studies, registry analyses (including Extracorporeal Life Support Organization (ELSO) data), experimental studies with translational relevance and international consensus statements and major guideline-related documents addressing ECPR. Case reports and small case series were not systematically included in the evidence synthesis, but were exceptionally considered when providing relevant mechanistic, technical or illustrative clinical insights.

Given the heterogeneity of the available evidence and the system-dependent nature of ECPR, a quantitative synthesis was not performed. Study selection and interpretation were performed through iterative consensus discussion among the authors, with emphasis placed on methodological quality, clinical relevance and applicability to the contemporary ECPR system of care. Studies were selected based on a hierarchical evaluation of evidence, prioritizing randomized controlled trials, followed by registry data, observational studies and expert consensus, while also considering methodological quality and clinical relevance. Particular attention was given to key domains central to ECPR implementation, including patient selection, the timing of initiation, pathophysiological mechanisms, complications, organizational models and ethical considerations. The selection process was guided by the objective of constructing a coherent conceptual framework linking biological plausibility, clinical evidence and healthcare system performance. Potential sources of bias were explicitly considered during interpretation. In particular, the review acknowledges the presence of selection bias, survival bias and confounding by system performance in observational and registry data, which may limit causal inference. Randomized trials were therefore interpreted within the context of implementation fidelity, organizational efficiency and low-flow duration, rather than in isolation. This approach was chosen to provide a clinically meaningful and integrative synthesis of a complex and evolving evidence base, rather than to provide a formal systematic review or quantitative meta-analysis.

## 3. Current Evidence and Mechanistic Insights

### 3.1. Pathophysiological Rationale for Extracorporeal Cardiopulmonary Resuscitation

Cardiac arrest represents an abrupt transition from organized circulation to systemic circulatory collapse, leading to global ischemia and rapid metabolic deterioration. The duration and severity of the no-flow and low-flow states are the primary determinants of irreversible organ injury [17,18]. Conventional cardiopulmonary resuscitation (CPR), although capable of generating partial forward flow, typically provides only 20–30% of normal cardiac output. This limited perfusion is often insufficient to sustain myocardial and cerebral metabolic demands, particularly beyond the first minutes of arrest [2].

#### 3.1.1. The Low-Flow State: Systemic Ischemia and Metabolic Collapse

During prolonged CPR, inadequate coronary perfusion pressure compromises myocardial oxygen delivery, aggravating ischemia-induced contractile dysfunction. Simultaneously, cerebral blood flow becomes highly dependent on compression quality and systemic vascular resistance, frequently falling below the thresholds required to preserve neuronal viability. At the cellular level, systemic hypoperfusion leads to mitochondrial dysfunction, including mitochondrial permeability transition pore (mPTP) opening, impaired oxidative phosphorylation and ATP depletion, as well as intracellular calcium overload mediated by Na^+^/H^+^ exchanger activation and subsequent Na^+^/Ca^2+^ exchanger reversal [19,20,21,22]. Anaerobic metabolism results in progressive lactate accumulation and metabolic acidosis, further impairing myocardial contractility and vascular tone. The ensuing ischemia–reperfusion cascade, characterized by reactive oxygen species generation, including succinate-driven ROS burst during reperfusion, endothelial dysfunction and inflammatory mediator release, contributes to post-resuscitation myocardial stunning, vasoplegia and multiorgan injury [6,23,24]. In this context, refractory cardiac arrest may be understood as a state of time-dependent metabolic collapse in which conventional CPR delays, but does not reverse, progressive organ injury.

#### 3.1.2. Extracorporeal Restoration of Systemic Perfusion

**Figure 1:**
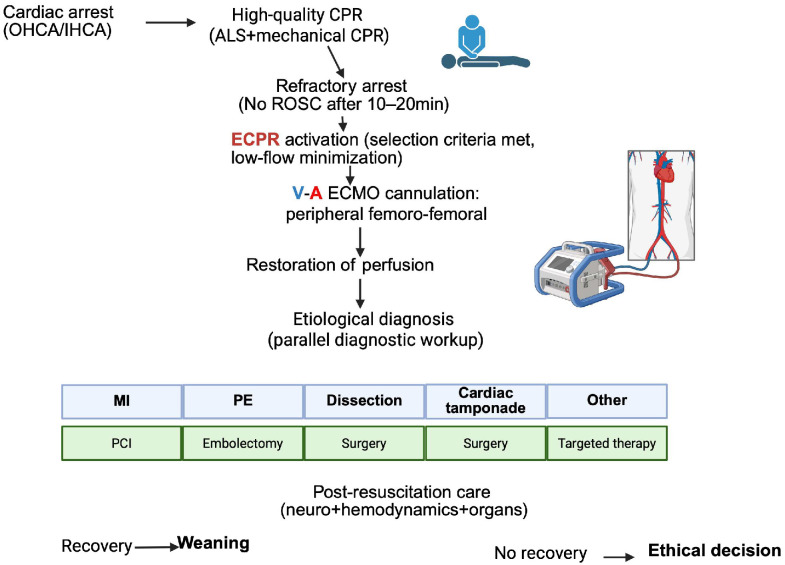
Conceptual framework of extracorporeal cardiopulmonary resuscitation (ECPR). Created in BioRender. Pirri, C. (2026) https://BioRender.com/bwq40w2.

Extracorporeal cardiopulmonary resuscitation (ECPR) directly addresses the central limitation of conventional CPR: insufficient organ perfusion. The rapid initiation of veno-arterial extracorporeal membrane oxygenation (V-A ECMO) restores near-normal systemic blood flow, augments coronary perfusion pressure, enables controlled oxygen delivery and facilitates carbon dioxide removal. By converting an unstable low-flow state into controlled extracorporeal circulation, ECPR may interrupt the downward spiral of metabolic acidosis, myocardial dysfunction and systemic inflammation [25,26,27,28]. The restoration of adequate perfusion provides a physiological bridge during which reversible causes, such as acute coronary occlusion, pulmonary embolism, severe electrolyte imbalance or toxin exposure can be identified and treated [29]. This shift reframes cardiac arrest not solely as an electrical event but as a profound, yet potentially reversible, phase of circulatory collapse. This conceptual transition is illustrated in Figure 1, which summarizes the shift from low-flow metabolic collapse to controlled extracorporeal perfusion (Figure 1).

#### 3.1.3. Myocardial Dysfunction and Reversible Stunning

Transient post-arrest myocardial dysfunction frequently contributes to early hemodynamic instability after cardiac arrest. In some patients, this dysfunction reflects reversible myocardial stunning secondary to global ischemia–reperfusion injury; however, in patients with extensive myocardial necrosis, severe multivessel coronary artery disease or non-revascularizable myocardial infarction, ventricular dysfunction may predominantly reflect irreversible structural injury rather than true stunning. Even when defibrillation is successful, global ischemia frequently results in severe systolic dysfunction that persists despite the restoration of organized electrical activity. V-A ECMO supports systemic perfusion independently of native cardiac output, thereby unloading the metabolic demand on a stunned myocardium while maintaining end organ circulation [30]. In patients with ischemic etiologies, extracorporeal support can stabilize hemodynamics long enough to allow for definitive revascularization or targeted therapy. However, this physiological benefit is time-sensitive. Prolonged no-flow intervals or extensive myocardial necrosis may limit the potential for recovery, underscoring the importance of early deployment within a biological salvageable window [29,31]. At the cellular level, myocardial stunning is associated with impaired sarcoplasmic reticulum calcium handling, including reduced SERCA2a activity and altered phospholamban phosphorylation, leading to impaired calcium reuptake. In addition, transient beta-adrenergic desensitization and persistent ATP depletion further contribute to impaired contractile function [32,33].

#### 3.1.4. Hemodynamic Interactions and Ventricular Loading

Although V-A ECMO restores systemic perfusion, it fundamentally alters cardiovascular loading conditions. Peripheral arterial return generates retrograde aortic flow, increasing left ventricular (LV) afterload. In the setting of severely depressed myocardial contractility, this rise in afterload may impair LV ejection and promote progressive ventricular distension. If the aortic valve fails to open due to insufficient contractile force, intracavitary blood stasis may develop, with potential consequences for myocardial recovery and intracardiac thrombosis. Thus, ECPR introduces a physiological paradox: while rescuing systemic circulation, it may simultaneously create a loading environment that challenges intrinsic ventricular recovery. This interaction between extracorporeal flow and native cardiac function is a defining hemodynamic feature of V-A ECMO support [34].

#### 3.1.5. Cerebral Perfusion and Neurological Viability

Neurologic injury remains the principal determinant of meaningful survival after cardiac arrest. Under conventional CPR, cerebral perfusion is variable and frequently inadequate, especially during prolonged low-flow intervals. By restoring continuous systemic flow, ECPR may preserve cerebral oxygen delivery and reduce secondary ischemic injury. Registry data indicate that a substantial proportion of survivors treated with ECPR achieve favorable neurological outcomes, suggesting that extracorporeal support may maintain neuronal viability when instituted early. Nevertheless, cerebral salvage remains strongly dependent on minimizing the duration of untreated ischemia [7,19,30].

#### 3.1.6. Differential Oxygenation: The Interaction Between Cardiac and Pulmonary Recovery

Peripheral femoro-femoral V-A ECMO also creates complex interactions between extracorporeal and native circulatory flows. As myocardial function recovers in the presence of persistent pulmonary dysfunction, oxygen-poor blood ejected from the left ventricle may compete with the retrograde oxygenated ECMO flow within the aorta. The position of the mixing point between these flows depends on the balance between native cardiac output and extracorporeal support. When native cardiac function improves while the lung function remains impaired, the proximal aortic arch, coronary and cerebral circulations may be preferentially perfused with less oxygenated blood. This phenomenon, known as differential oxygenation (or Harlequin syndrome), illustrates the dynamic interplay between cardiac recovery and pulmonary injury in the post-arrest state [35].

#### 3.1.7. From Resuscitation to Controlled Extracorporeal Physiology

ECPR represents more than mechanical support; it constitutes a transition from intermittent compressive perfusion to regulated extracorporeal physiology. Continuous flow, controlled gas exchange and the real-time correction of metabolic abnormalities allow clinicians to stabilize the internal milieu addressing the underlying cause of arrest [25]. However, physiological rescue is contingent upon timing. If instituted too late, extracorporeal support may merely sustain already irreversible injury. Thus, the pathophysiological rationale for ECPR rests on a narrow therapeutic window in which systemic perfusion can be restored before critical thresholds of organ damage are exceeded.

### 3.2. Evidence from Observational Studies and Registries

The early clinical experience with extracorporeal cardiopulmonary resuscitation (ECPR) was largely derived from single-center case series and registry-based analyses. These observational data provided the first signals that extracorporeal support could improve survival in carefully selected patients with refractory cardiac arrest [36,37]. Among adult patients recorded in the Extracorporeal Life Support Organization (ELSO) registry, survival to hospital discharge approximates 29%. Importantly, a substantial proportion of survivors demonstrate favorable neurological outcomes, often categorized as Cerebral Performance Category (CPC) 1–2 [8]. These findings suggest that extracorporeal support might not only increase survival, but also preserve meaningful neurological recovery. Multiple institutional cohorts have subsequently reported survival rates ranging from 15% to 50% depending on the patient selection, timing of cannulation and system organization [7,38]. Higher survival rates have consistently been observed in witnessed arrests, short no-flow intervals, initial shockable rhythms, presumed reversible etiologies, and high-volume ECMO centers [39]. However, these encouraging results must be interpreted cautiously. Observational studies are inherently susceptible to selection bias, survival bias and confounding by system performance. Patients chosen for ECPR are often younger, more likely have shockable rhythms and treated in highly specialized centers with rapid deployment protocols. Thus, improved outcomes may reflect not only the device itself but also the surrounding systems of care. Furthermore, registry analyses demonstrate marked heterogeneity in cannulation timing, inclusion criteria and post-resuscitation management strategies. This variability limits generalizability and underscores that ECPR outcomes are tightly linked to organizational efficiency. Indeed, volume–outcome relationships suggest that high-expertise centers achieve superior results. Importantly, these data do not establish a causal relationship between ECPR and improved outcomes. Rather, they likely reflect a combination of patient selection, intervention timings and system-level efficiency. As such, observational findings should be interpreted as hypothesis-generating and context-dependent, rather than as definitive evidence of treatment effect.

### 3.3. Randomized Controlled Trials

Only recently have randomized controlled trials (RCTs) begun to evaluate whether the physiological advantages of ECPR translate into improved survival compared with conventional CPR alone [11,12,13]. Several landmark trials shaped the current debate (Table 1).

- ARREST Trial

A single-center randomized study of refractory ventricular fibrillation OHCA demonstrated significantly higher survival to hospital discharge with favorable neurological outcome in the ECPR arm compared to standard advanced cardiac life support. The trial was stopped early due to apparent benefit. Importantly, it was conducted within a highly organized regional resuscitation system with rapid cath-lab cannulation and strict inclusion criteria [11].

- Prague OHCA Trial

This multicenter trial evaluated a bundled invasive strategy including ECPR, early transport and immediate coronary angiography. Although not statistically superior in the primary analysis, adjusted analyses suggested a benefit in selected subgroups, particularly those with shockable rhythms and shorter low-flow times [12].

- INCEPTION Trial

In contrast, this multicenter randomized trial did not demonstrate a statistically significant difference in 30-day survival with favorable neurological outcome between ECPR and conventional CPR. Delays in cannulation and system heterogeneity were notable features [13]. Overall, these trials suggest that ECPR outcomes are strongly influenced by timing, organizational efficiency and rapid access to definitive strategy.

Importantly, most randomized data remain limited to patients with refractory shockable rhythms, whereas the role of ECPR in non-shockable rhythms, unwitnessed arrest or prolonged ischemic intervals remains uncertain. A critical interpretation of these trials suggests that they do not simply compare ECPR versus conventional CPR, but rather reflect differences between optimized and variable-quality resuscitation systems. The ARREST trial may be interpreted as a proof-of-concept of ECPR efficacy under highly controlled conditions, characterized by rapid cannulation, strict patient selection and immediate access to definitive therapy. In contrast, the INCEPTION trial reflects the challenges of implementing ECPR across heterogeneous systems, where delays in cannulation and variability in expertise may attenuate potential benefit. The Prague OHCA trial occupies an intermediate position, combining elements of system integration with real-world logistical constraints. Taken together, these findings reinforce the concept that the effectiveness of ECPR is not solely determined by the technology itself, but by the ability of the surrounding system to minimize low-flow duration and ensure rapid transition to definitive treatment. Thus, randomized evidence should be interpreted within the context of system performance, rather than as an isolated comparison between treatment modalities.

Low-flow duration values are approximate and derived from published trial population; they are reported to improve contextual interpretation of system-related differences across studies.

### 3.4. Patient Selection and Timing

The effectiveness of ECPR is inseparable from appropriate patient selection and rapid initiation [25,33]. Unlike conventional CPR, which is universally applied, ECPR is a resource-intensive intervention requiring the selective deployment within a narrow therapeutic window.

#### 3.4.1. Patient Selection

Current consensus recommendations emphasize the identification of patients most likely to achieve neurologically meaningful recovery. Across observational data and randomized trials, several variables consistently correlate with improved outcomes:

- witnessed cardiac arrest [40];
- immediate bystander CPR (no-flow < 5 min) [25,40];
- initial shockable rhythm; presumed reversible etiology [41,42];
- age below 65–70 years (relative criterion) [43];
- absence of major comorbidities [25];
- short anticipated low-flow duration (<60 min) [25,44].

Shockable rhythms (ventricular fibrillation or pulseless ventricular tachycardia) demonstrate the strongest association with benefit, likely reflecting underlying ischemic mechanisms amenable to rapid coronary intervention [37]. Conversely, non-shockable rhythms, unwitnessed, prolonged no-flow intervals and severe pre-existing comorbidity significantly reduce the probability of favorable neurological outcome [25,40,41,42,43,44]. While ECPR has occasionally been used in these contexts, the balance between potential benefits and futility becomes increasingly uncertain. Observational and registry data suggest that survival with good neurological outcome in ECPR-treated patients is approximately 25–30% overall, but this probability varies substantially according to clinical context. Favorable profiles, characterized by witnessed arrest, immediate bystander CPR (no-flow < 5 min), initial shockable rhythms and low-flow duration below 60 min, are consistently associated with higher survival rates in observational series and randomized trials populations. In contrast, prolonged low-flow duration (>60–90 min), non-shockable rhythms, unwitnessed arrest and severe comorbidities are associated with markedly reduced likelihood of meaningful recovery (with survival rates below 10%) [25,38,41,42]. Importantly, these figures should be interpreted as context-dependent rather than as absolute thresholds. Current evidence does not support rigid cut-off values; instead, outcome probability reflects the cumulative interaction between timing, physiology and system performance.

In clinical practice, patient selection must occur under conditions of incomplete information and extreme time pressure. For this reason, most high-performing systems rely on predefined activation criteria embedded within resuscitation protocols rather than ad hoc clinical judgment (Table 2).

#### 3.4.2. Timing: The Low-Flow Imperative

**Figure 2:**
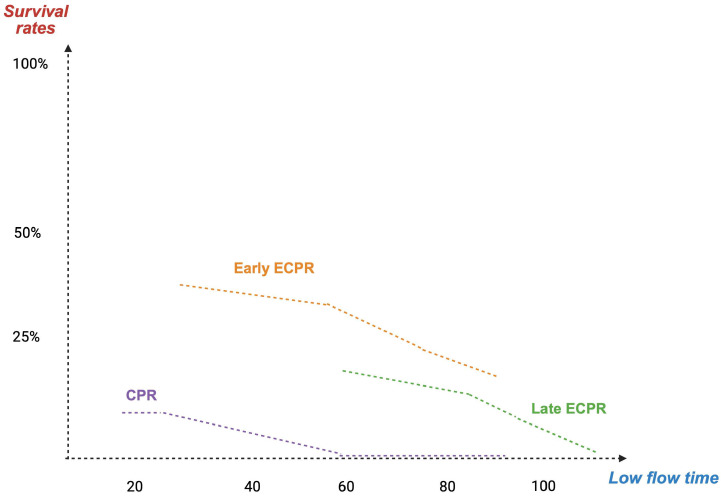
Time-dependent relationship between low-flow duration and survival in ECPR. ECPR: extracorporeal cardiopulmonary resuscitation; CPR: cardiopulmonary resuscitation. Created in BioRender. Pirri, C. (2026) https://BioRender.com/ib8btd2.

As shown in Figure 2, the probability of favorable outcomes declines sharply as low-flow duration increases, reinforcing the time-sensitive nature of ECPR (Figure 2). The interval between cardiac arrest and the establishment of effective extracorporeal flow strongly influences neurological outcome. Observational studies suggest a marked decline in survival beyond 60 min of low-flow duration [11,45,46,47].

ECPR therefore requires a balance between premature escalation and delayed intervention, as prolonged low-flow duration rapidly reduces the likelihood of meaningful recovery. High-performance systems minimize this window through the early identification of refractory arrest (typically after 10–15 min of failed advanced life support), prehospital notification and activation, parallel preparation during ongoing CPR, and immediate access to cannulation expertise [48]. However, the optimal activation threshold may vary according to local system capability, including transport logistics, cannulation readiness, team availability and the expected time-to effective perfusion.

### 3.5. Cannulation Strategy and Technical Considerations

In adult refractory cardiac arrest, ECPR is almost universally established through percutaneous peripheral femoro-femoral cannulation. This configuration has become the operational standard not because it is physiologically ideal, but because it is logistically compatible with ongoing cardiopulmonary resuscitation [29]. The defining constraint during arrest is the need to maintain uninterrupted chest compressions while preparing for extracorporeal support [49]. Percutaneous femoral access allows for vascular cannulation without thoracotomy, without patient repositioning and without discontinuing resuscitative efforts [50,51]. Its feasibility during mechanical CPR has been a decisive factor in the expansion of ECPR programs. Distal perfusion cannula placement is widely considered standard practice to reduce the risk of limb ischemia. In adult ECPR, target extracorporeal flow is typically maintained in the range of 60–80 mL/kg/min to ensure adequate systemic perfusion. Serial lactate clearance may serve as a useful surrogate marker of perfusion adequacy and metabolic recovery [52,53,54].

#### 3.5.1. Procedural Environment and Workflow

The practical execution of percutaneous cannulation depends heavily on workflow standardization. Successful programs typically employ parallel task allocation: while high-quality CPR continues, one team prepares the groin fields, establishes ultrasound-guided vascular access and advances guidewires; another team manages airway and pharmacologic support; and a third coordinates ECMO and circuit priming. Ultrasound guidance has become essential in this context, reducing failed access attempts and minimizing vascular injury, particularly in patients with vasoconstriction or obesity. The emphasis in ECPR cannulation is therefore not on technical variation, but on procedural reliability under extreme time pressure [51,55].

#### 3.5.2. Vascular Access and Flow Adequacy

The femoral vessels provide rapid access to large-caliber conduits capable of sustaining high extracorporeal flow. Adequate venous drainage is particularly critical in the arrest setting, as extracorporeal circulation replaces essentially all native cardiac output. Cannula sizing and positioning must therefore be sufficient to achieve flows capable of restoring systemic perfusion. Unlike elective ECMO initiation, where partial native cardiac output may persist, ECPR requires the immediate establishment of fully circulatory support to reverse ongoing global ischemia and metabolic deterioration. The cannulation strategy in this context is thus directly linked to the capacity to deliver full circulatory support from the outset [29,55,56,57,58].

#### 3.5.3. Operational Constraints

Cardiac arrest presents additional challenges not encountered in other ECMO scenarios. Profound vasoconstriction, hypovolemia, ongoing compressions and limited patient history increase technical difficulty. Moreover, the decision-making occurs in the absence of complete diagnostic clarity. Cannulation must therefore proceed under conditions of uncertainty, with simultaneous diagnostic and therapeutic intent. For these reasons, procedural expertise and rehearsal are central to ECPR readiness. The technical act of cannulation is embedded within a predefined activation protocol rather than initiated as an improvised intervention [59,60].

### 3.6. Etiological Diagnosis and Definitive Treatment Pathways During ECPR

ECPR should not be conceived as an endpoint intervention, but as a bridge to definitive diagnosis and treatment. The restoration of systemic perfusion through V-A ECMO creates a narrow therapeutic window during which the underlying cause of cardiac arrest must be rapidly identified and, when reversible, corrected. Failure to clarify etiology risks transforming extracorporeal support into a bridge to nowhere strategy (Table 3).

#### 3.6.1. Parallel Stabilization and Diagnostic Workflow

Etiologic investigation during ECPR must proceed in parallel with hemodynamic stabilization. The diagnostic approach is guided by three principles:

(1) Time sensitivity: Potentially reversible causes require immediate identification;
(2) Probability-based prioritization: Diagnostic pathways should reflect the epidemiology of arrest (e.g., ischemic causes in shockable rhythms);
(3) Integration with definitive therapy: Diagnostic steps should be embedded within actionable treatment pathways.

Point-of-care tools (arterial blood gas analysis, lactate trends, and bedside echocardiography) provide immediate physiologic orientation, while advanced imaging and invasive diagnostics follow once circulatory stability is achieved [61,62,63,64,65].

#### 3.6.2. Coronary Occlusion and Acute Myocardial Infarction

In adults presenting with refractory shockable rhythms, acute coronary occlusion represents a leading etiology. Early coronary angiography and percutaneous coronary intervention (PCI) are therefore central components of many ECPR pathways. ECPR programs that integrate rapid cannulation with immediate transfer to the cardiac catheterization laboratory allow simultaneous mechanical support and causal correction. Even in the absence of ST-segment elevation, a coronary cause may be present and diagnostic coronary angiography is frequently considered in the absence of an alternative explanation [11,62,66,67].

#### 3.6.3. Pulmonary Embolism

Massive pulmonary embolism (PE) should be suspected in cases with preceding dyspnea, right ventricular dilation on echocardiography or unexplained pulseless electrical activity. Bedside transthoracic or transesophageal echocardiography may reveal right ventricular strain, septal flattening or right-sided thrombus in transit [63]. When PE is strongly suspected, in the absence of extracorporeal support, systemic thrombolysis is often considered emergently given the high mortality associated with persistent circulatory collapse [68]. However, once ECPR has been established and systemic perfusion restored, the therapeutic landscape changes. The presence of recent chest compressions, vascular cannulation and mandatory anticoagulation significantly increases hemorrhagic risk, particularly for intrathoracic bleeding. In this context, ECMO may provide circulatory stabilization, allowing for the consideration of surgical embolectomy or catheter-directed intervention as an alternative to systemic thrombolysis [69,70]. The choice among these strategies should be guided by institutional expertise, bleeding risk assessment and the overall neurological and hemodynamic profile of the patient.

#### 3.6.4. Structural and Mechanical Causes

Pericardial tamponade, acute aortic pathology and mechanical complications of myocardial infarction require prompt exclusion during ECPR [62]. Focused echocardiography is critical in identifying tamponade, severe valvular dysfunction, ventricular septal rupture or acute papillary muscle failure [63,71]. In selected hemodynamically stable patients supported with ECMO, computed tomography may be performed to evaluate suspected aortic dissection or intracranial pathology, provided that transport can be accomplished safely [72,73]. Particular caution is warranted when acute aortic dissection is identified after the initiation of peripheral V-A ECMO. In the case of Stanford A type dissection, retrograde aortic flow may increase wall stress in the ascending aorta, potentially exacerbating propagation of the dissection flap, worsening aortic regurgitation or compromising coronary perfusion. In such scenarios, extracorporeal support must be rapidly reframed as a bridge to emergent surgical repair when feasible [74]. In this setting, cannulation strategy becomes particularly important. Peripheral femoral arterial cannulation may be problematic because retrograde ECMO flow can increase false lumen pressurization and potentially aggravate malperfusion syndromes. In selected surgical candidates, alternative cannulation approaches such as axillary or subclavian cannulation may provide more physiological antegrade perfusion and may improve cerebral and coronary blood flow. In patients proceeding directly to operative repair, central cannulation may also be considered when surgically feasible. Therefore, in acute type A aortic dissection, the choice of cannulation site should be individualized according to aortic anatomy, malperfusion pattern, anticipated surgical strategy and institutional expertise [75,76,77].

Conversely, in patients deemed non-surgical candidates because of extensive neurologic injury or prohibitive surgical risk, the continuation of ECMO support should be carefully reconsidered in light of overall prognosis.

#### 3.6.5. Primary Electrical Disorders and Channelopathies

In the absence of structural or ischemic pathology, primary arrhythmic syndromes should be considered, particularly in younger patients. Electrocardiographic patterns suggestive of channelopathies (e.g., long QT syndrome; Brugada pattern) may guide further management and long-term planning, although therapeutic implications are often limited [78,79].

#### 3.6.6. Metabolic and Toxicological Causes

Electrolyte disturbances, severe hypoxia, toxin exposure or drug overdose may underlie refractory arrest. Rapid laboratory assessment, including potassium, arterial blood gases, toxicology screening and lactate, can orient management toward targeted correction or antidotal therapy [80,81,82].

#### 3.6.7. Diagnostic Uncertainty and Decision-Making

Despite systematic evaluation, a proportion of patients will have indeterminate etiology. In such cases, continued support must be guided by dynamic assessment of organ recovery, neurological status and likelihood of reversibility.

The diagnostic process during ECPR is therefore iterative rather than linear. Stabilization enables diagnosis; diagnosis guides definitive therapy; and response to therapy informs prognosis.

#### 3.6.8. Bridge to Decision, Bridge to Transplant and Bridge to Durable Mechanical Support

In selected patients who do not demonstrate early myocardial recovery after ECPR, extracorporeal support may function as a bridge to decision strategy. This period allows clinicians to evaluate neurological prognosis, the reversibility of myocardial dysfunction, end organ recovery and candidacy for advanced heart failure therapies. In patients with irreversible myocardial injury but preserved neurological potential, transition from temporary extracorporeal support to durable mechanical circulatory support or heart transplantation may be considered. In this context, ECPR should not be regarded as definitive therapy, but rather as an initial stabilization platform within a broader advanced heart failure pathway. Bridge to transplant strategies may be appropriate in carefully selected patients without irreversible multiorgan failure or severe neurological injury. Similarly, bridge to durable left ventricular assist device (LVAD) support may be considered in patients with persistent severe ventricular dysfunction who are unlikely to recover native cardiac function but remain suitable candidates for long-term mechanical circulatory support. However, these strategies require complex multidisciplinary assessment involving intensivists, heart failure specialists, cardiac surgeons, neurologists and transplant teams. Careful patient selection remains essential because prolonged extracorporeal support in the absence of realistic recovery or advanced therapy candidacy may increase morbidity without improving meaningful survival [77,83].

### 3.7. Complications and Post-Resuscitation Management

The initiation of cardiopulmonary resuscitation marks only the beginning of a complex phase of critical care. While veno-arterial extracorporeal membrane oxygenation restores systemic perfusion, it simultaneously introduces physiological perturbations and procedure-related risks that may influence survival and neurological recovery. Thus, the benefit of ECPR depends not only on rapid cannulation but on meticulous post-resuscitation management (Table 4) [84].

#### 3.7.1. Left Ventricular Distension

In a subset of patients, the altered loading conditions inherent to peripheral V-A ECMO may become clinically significant. When native myocardial recovery is delayed, impaired aortic valve opening and progressive ventricular dilatation can compromise pulmonary function and impede myocardial recovery. This may be associated with increased pulmonary capillary wedge pressure [85,86]. Management strategies are therefore directed toward promoting ventricular unloading and restoring effective ejection. These may include the modulation of extracorporeal flow, inotropic support to facilitate aortic valve opening and the use of adjunctive unloading devices such as intra-aortic balloon pump (IABP) or percutaneous ventricular assist systems (e.g., Impella). In selected cases, direct venting strategies may be required (Table 5). The goal is not merely hemodynamic optimization, but preservation of myocardial recovery potential [86,87,88].

#### 3.7.2. Differential Oxygenation

The flow interaction described above may translate into clinically significant upper-body hypoxemia once native cardiac function partially recovers in the presence of severe pulmonary dysfunction. When poorly oxygenated blood is ejected from the left ventricle, it may preferentially perfuse the aortic root and proximal great vessels before mixing with the retrograde oxygenated ECMO flow. Because coronary arteries originate from the aortic root and cerebral vessels arise from the aortic arch, inadequate monitoring may result in unrecognized hypoxic exposure of both myocardial and cerebral territories despite apparently satisfactory systemic oxygenation [35,85]. Management focuses on optimizing native lung function through ventilatory adjustment, recruiting strategies and correction of reversible pulmonary pathologies. In cases where differential hypoxia persists despite maximal ventilatory support, the modification of extracorporeal configuration, such as the adjustment of the flow balance or the conversion to hybrid configurations, may be considered to ensure adequate cerebral oxygen delivery [89,90,91]. In persistent differential hypoxia, hybrid extracorporeal configurations may be required. Veno-arterial-venous (VAV) ECMO can be established by adding a venous return cannula, typically in the right internal jugular vein, allowing oxygenated blood to be delivered both to the arterial circulation and to the right atrium. This strategy increases oxygen content in the pulmonary circulation and improves oxygenation of blood ejected by the recovering left ventricle, thereby reducing hypoxemia of the coronary and cerebral territories. Conversely, in patients with substantial myocardial recovery but persistent severe respiratory failure, conversion from V-A ECMO to VV ECMO may be considered. In this setting, extracorporeal support no longer primarily serves circulatory assistance but rather respiratory support while native cardiac output becomes sufficient to maintain systemic perfusion. Therefore, the management of Harlequin syndrome should be individualized according to the dynamic interaction between myocardial recovery, pulmonary function and extracorporeal flow distribution [35,92].

#### 3.7.3. Vascular and Limb Complications

Percutaneous femoral cannulation, although rapid and effective, carries inherent vascular risks. Arterial obstruction, distal hypoperfusion, hematoma and vessel injury may occur, particularly in the context of vasoconstriction or prolonged low-flow states. Limb ischemia remains a clinically significant complication in peripheral V-A ECMO. The regular assessment of distal perfusion, Doppler evaluation and early intervention are critical to prevent irreversible ischemic injury. Vascular complications may substantially affect morbidity even in otherwise neurologically favorable survivors [93,94].

#### 3.7.4. Hemorrhage and Coagulation Disorders

Bleeding is among the most frequent complications during ECPR. Several mechanisms converge in this setting: traumatic vascular access during active CPR, compression-related thoracic injuries, systemic anticoagulation, platelet dysfunction and coagulopathy associated with cardiac arrest. Acquired von Willebrand syndrome may further contribute to bleeding risk during ECMO support. High shear stress generated within the extracorporeal circuit promotes the conformational unfolding and proteolytic cleavage of high-molecular weight von Willebrand multimers by ADAMTS 13, resulting in impaired platelet adhesion and defective primary hemostasis. This ECMO-associated loss of functional von Willebrand factor may contribute to mucosal, surgical and cannulation-site bleeding, particularly in the presence of systemic anticoagulation and ECMO-induced platelet dysfunction [95].

The balance between preventing circuit thrombosis and minimizing hemorrhagic complications is particularly delicate during the early post-resuscitation phase [96,97,98,99,100]. Major bleeding events, including intracranial hemorrhage, may negate the survival benefit of extracorporeal support and complicate neurological prognostication [101].

Unfractionated heparin remains the standard anticoagulant during ECMO because of its familiarity, reversibility and relatively low cost. However, direct thrombin inhibitors, particularly bivalirudin, have emerged as alternative anticoagulation strategies in selected patients. Bivalirudin may be particularly useful in the presence of heparin-induced thrombocytopenia, heparin resistance, unstable anticoagulation profiles or recurrent circuit thrombosis despite apparently adequate heparinization. Unlike heparin, bivalirudin directly inhibits both circulating and clot-bound thrombin and provides more predictable pharmacokinetics with reduced dependence on antithrombin activity. Some observational studies have suggested lower rates of circuit thrombosis, reduced transfusion requirements and more stable anticoagulation parameters with bivalirudin-based strategies. Nevertheless, its use remains limited by higher cost, the absence of a specific reversal agent and the lack of robust randomized evidence in ECPR populations. Therefore, while unfractionated heparin remains the most widely adopted anticoagulant during ECPR, alternative anticoagulation strategies should be individualized according to bleeding risk, thrombotic complications, heparin tolerance and institutional experience [102,103].

#### 3.7.5. Neurological Management and Prognostication

Neurological outcome remains the ultimate determinant of meaningful survival after cardiac arrest. Although ECPR restores cerebral perfusion, it does not reverse established hypoxic–ischemic injury. Post-resuscitation neurocritical care is therefore central to translating circulatory stabilization into functional recovery [104,105].

Early management focuses on minimizing secondary brain injury through the strict control of oxygenation, ventilation, temperature, perfusion pressure and metabolic homeostasis. Both hypoxemia and hyperoxia may exacerbate oxidative stress and neuronal injury; similarly, extreme carbon dioxide tension may alter cerebral blood flow autoregulation [6,17,106,107,108]. Targeted temperature management (TTM) remains an important component of care, particularly in comatose patients, although its optimal temperature target in the context of ECPR remains debated. The maintenance of adequate mean arterial pressure is essential to preserve cerebral perfusion, especially given the potential alterations in autoregulatory capacity following prolonged low-flow states. Continuous hemodynamic monitoring and the avoidance of hypotension are therefore critical during the first 24–72 h [17,106,108,109,110,111,112,113,114].

Electrographic seizures and non-convulsive status epilepticus are common after cardiac arrest and may occur without overt clinical manifestations. Continuous or intermittent electroencephalographic monitoring is recommended when available [17,102,106,107]. Sedation strategies should balance patient comfort and ventilatory synchrony with the need for periodic neurological assessment. In ECPR patients, sedation is often deeper and more prolonged due to extracorporeal support and mechanical ventilation, complicating neurological evaluation. The presence of ECMO-related metabolic disturbances, hypothermia or organ dysfunction further confounds early clinical assessment [115,116,117].

Accurate neurological prognostication is particularly challenging in the ECPR population. The premature withdrawal of life-sustaining therapy based on early, unreliable markers may preclude recovery in potentially salvageable patients [117]. A multimodal approach, integrating clinical examination, electrophysiology, neuroimaging and biomarkers, is recommended. Neuron-specific enolase (NSE) and S100B may support prognostication within a multimodal framework, although interpretation in ECPR patients is complicated by hemolysis, renal dysfunction and extracorporeal circulation [118,119,120,121].

Neuroimaging may provide additional prognostic information. Brain computed tomography may reveal early signs of severe hypoxic–ischemic injury, including the loss of gray–white matter differentiation and diffuse cerebral edema. In selected patients, brain magnetic resonance imaging demonstrates diffusion-weighted imaging (DWI) patterns consistent with diffuse hypoxic–ischemic injury may further support neurological prognostication. The extent and distribution of diffusion restriction may help support neurological prognostication when integrated within a multimodal assessment framework [122,123].

Importantly, prognostic evaluation should be delayed until confounding factors such as sedation, hypothermia and metabolic abnormalities are resolved [104,124].

Registry data indicate that a substantial proportion of survivors treated with ECPR achieve favorable neurological outcomes, underscoring the need for cautious and structured prognostic strategies [7,8,125,126]. Thus, neurological management in ECPR extends beyond supportive care; it represents a critical determinant of outcome and requires specialized neurocritical expertise.

#### 3.7.6. Multiorgan Support

- Acute kidney injury and CRRT integration

Refractory cardiac arrest and subsequent reperfusion frequently precipitate a systemic inflammatory and ischemia–reperfusion response affecting multiple organs. Even after the restoration of circulatory stability with ECPR, patients often exhibit acute kidney injury (AKI), hepatic dysfunction, vasoplegia and metabolic derangements [127,128,129,130].

Acute kidney injury is common following cardiac arrest and is associated with increased mortality. Mechanisms include prolonged hypoperfusion, reperfusion injury, systemic inflammation and exposure to vasoactive agents [128,131]. In the context of V-A ECMO, renal dysfunction may be exacerbated by hemolysis, inflammatory activation and fluid shifts [132,133]. Continuous renal replacement therapy (CRRT) is frequently employed to manage volume overload, electrolyte disturbances and acid–base imbalance. CRRT may be delivered either through integration within the ECMO circuit or via a dedicated vascular access, depending on institutional practice and technical considerations. Early initiation may facilitate controlled fluid balance, reduce interstitial edema and contribute to hemodynamic stability. When integrated into the extracorporeal circuit, careful coordination is required to avoid circuit instability, excessive negative pressures or unintended alteration in flow dynamics [134]. Although robust randomized data are limited, observational experience suggests that proactive renal support may improve fluid management and organ recovery in selected patients [135].

- Systemic inflammation and vasoplegia

Post-arrest syndrome is characterized by a sepsis-like inflammatory response, including cytokine release, endothelial dysfunction and vasoplegia. This response is characterized by a cytokine cascade involving interleukin-6 (IL-6), tumor necrosis factor-α, interleukin-1 β (IL-1β), contributing to endothelial dysfunction, glycocalyx degradation and microcirculatory alterations. These process may result in persistent microcirculatory shunting despite the restoration of macrocirculatory flow, partially explaining the dissociation between hemodynamic stabilization and tissue-level recovery [30,130]. Even under full extracorporeal support, patients may require substantial vasopressor therapy to maintain adequate perfusion pressure. Management focuses on balancing vasopressors support with adequate preload, minimizing fluid overload and correcting metabolic disturbances [129]. In selected cases, adjunctive therapies targeting inflammatory pathways have been explored, such as hemoadsorption techniques or pharmacologic vasoplegia-targeted therapies, although definitive evidence remains limited [136,137,138].

- Hepatic and metabolic dysfunction

Liver dysfunction following cardiac arrest reflects both hypoxic injury and systemic inflammatory response. Impaired lactate clearance may persist despite restored perfusion, complicating interpretation of metabolic recovery. The careful assessment of dynamic trends rather than isolated values is therefore essential in evaluating systemic improvement [129,139].

#### 3.7.7. Circuit-Related Complications and Extracorporeal System Durability

Beyond patient-related complications, ECPR is also associated with circuit-related adverse events, including hemolysis, thromboembolism, oxygenator dysfunction, circuit thrombosis, infection and air embolism. These complications become increasingly relevant as ECMO duration increases, and may substantially affect both extracorporeal support quality and end organ recovery. Hemolysis may result from excessive shear stress, high pump speeds, circuit thrombosis or excessive negative drainage pressures [140].

Elevated plasma-free hemoglobin may contribute to acute kidney injury, endothelial dysfunction and impaired oxygen delivery [141]. Oxygenator dysfunction may manifest as progressive transmembrane pressure increase, reduced gas exchange efficiency or visible thrombus formation, occasionally requiring urgent oxygenator or circuit exchange [142]. Infectious complications also represent a major concern during prolonged intensive care exposure: invasive monitoring and critical illness-associated immune dysregulation increases the risk of bloodstream infection and device-related colonization [143]. Air embolism, although uncommon in contemporary systems, remains a potentially catastrophic complication and requires meticulous circuit surveillance, secure connections and strict management protocols [144]. Modern ECMO system incorporate multiple safety systems incorporate multiple safety mechanisms, including pressure monitoring, bubble detectors and integrated flow surveillance, in order to reduce these risks. Contemporary ECMO systems differ in terms of pump technology, oxygenator membrane design, circuit biocompatibility, monitoring capability, reduced circuit thrombogenicity and facilitated deployment in emergency and transport settings. Modern centrifugal pumps are associated with lower hemolysis and improved flow stability compared with older roller pump system, whereas polymethylpentene oxygenator membranes provide more durable gas exchange performance and a lower plasma leakage risk. In addition, miniaturized and portable ECMO platforms have facilitated the development of mobile ECPR programs and interhospital transport strategies (Table 6). Therefore, extracorporeal system durability should be interpreted not only in terms of mechanical longevity, but also in relation to hemocompatibility, thrombotic resistance, monitoring reliability, portability and the capacity to sustain prolonged extracorporeal support with acceptable complication rates [145,146,147,148].

### 3.8. System of Care and Organizational Models

ECPR outcomes depend not only on extracorporeal technology, but also on the efficient organizational models capable of minimizing delays and integrating definitive treatment pathways [11,12,13].

#### 3.8.1. Structured Activation and Early Identification

High-performing ECPR programs rely on predefined activation criteria and the rapid identification of refractory cardiac arrest. Clear inclusion and exclusion parameters, such as witnessed arrest, limited no-flow time, shockable rhythm and presumed reversible etiology, enable early decision-making and minimize delays [61,149,150,151,152].

#### 3.8.2. Regionalization and Volume–Outcome Relationship

Registry analyses indicate that survival rates are higher in experienced, high-volume ECMO centers. This observation supports regionalized models in which selected patients with refractory arrest are transported to specialized centers capable of immediate cannulation and comprehensive post-resuscitation management. Regionalization may improve outcomes by concentrating expertise and infrastructure, although transport-related delays and equitable access remain important challenges [14,60,153].

#### 3.8.3. Integration with Definitive Treatment Pathways

ECPR must be embedded within pathways that provide immediate access to etiologic treatment. In ischemic arrest, rapid transition to coronary angiography and revascularization is central. In cases of suspected pulmonary embolism or mechanical complications, access to surgical or interventional expertise is required. Programs in which cannulation, diagnostic clarification and definitive intervention occur in rapid succession demonstrate more consistent outcome signals than those in which ECPR is implemented in isolation [11,61].

#### 3.8.4. Multidisciplinary Coordination and Simulation

Effective ECPR implementation demands collaboration among emergency physicians, cardiologists, cardiac anesthesiologists, intensivists, perfusionists, nurses and prehospital providers. Defined roles, simulation-based training and standardized workflows reduce procedural delays and technical errors [154,155].

#### 3.8.5. Prehospital and Mobile ECPR Models

Emerging models aim to initiate ECPR in prehospital settings to further reduce low-flow duration. While early data from highly specialized systems are promising, these approaches require substantial resource allocation and rigorous governance structures. Their scalability and cost-effectiveness remain uncertain [11,59].

#### 3.8.6. Resource Allocation and Sustainability

ECPR is resource-intensive, requiring specialized equipment, personnel and prolonged intensive care support. Organizational models must therefore consider sustainability, equitable access and opportunity cost. Transparent governance and continuous outcome monitoring are essential to ensure appropriate utilization [156].

Because ECPR requires highly specialized personnel, dedicated infrastructure and prolonged intensive care support, future studies should integrate formal cost-effectiveness analyses alongside survival and neurological outcomes in order to better define the sustainability of different organizational models.

### 3.9. Ethical and Resource Allocation Considerations

ECPR raises complex ethical and resource-related challenges that extend beyond traditional resuscitation paradigms. By transforming refractory cardiac arrest into a potentially reversible state of supported circulation, ECPR blurs the boundary between life-saving intervention and the prolongation of non-recoverable conditions. This raises the risk of a bridge to nowhere scenario, in which extracorporeal support is initiated without a realistic pathway to recovery. In this context, it is important to distinguish between physiological success and survival with meaningful neurological recovery. Consequently, decisions surrounding its initiation, continuation and withdrawal require the careful consideration of clinical benefit, patient values and system-level constraints [156,157,158].

#### 3.9.1. Patient Selection and the Risk of Futility

The ethical justification for ECPR is closely linked to the probability of achieving survival with meaningful neurological recovery [96]. While selection criteria aim to identify patients most likely to benefit, real-time decision-making often occurs under conditions of uncertainty, incomplete information and time pressure. Inappropriate application of ECPR in patients with prolonged no-flow time, severe comorbidities or non-reversible etiologies may result in physiological support without a realistic prospect of recovery [159,160]. In such cases, extracorporeal supports risks becoming a means of prolonging dying rather than restoring life. The concept of physiological success must therefore be distinguished from “clinical success”: restoration of circulation does not equate to meaningful survival. Operationally, futility in ECPR may be considered when extracorporeal support restores macrocirculation despite the absence of a realistic pathway toward neurological recovery, definitive etiologic treatment, durable circulatory support, transplantation or survival with acceptable functional outcome.

#### 3.9.2. Uncertainty and Time-Dependent Decision-Making

ECPR introduces a temporal paradox. Early initiation is necessary to preserve organ viability, yet comprehensive prognostic information, particularly neurological status, becomes available only later [104]. This creates a phase of “therapeutic uncertainty” in which aggressive support is continued despite unclear long-term prognosis. During this period, the premature limitation of care may preclude recovery, while prolonged support in non-recoverable patients may expose individuals to burdensome interventions without benefit. Structured reassessment at predefined time points, incorporating neurological, hemodynamic and etiological data, is therefore essential [109,161].

#### 3.9.3. Withdrawal of Extracorporeal Support

The decision to withdraw ECMO support represents one of the most ethically challenging aspects of ECPR. These decisions should be based on a comprehensive evaluation of neurological prognosis, the reversibility of the underlying cause, the degree of multiorgan failure, and the patient’s values and previously expressed wishes. Given the complexity of prognostication, especially under sedation and extracorporeal support, withdrawal decisions should be multidisciplinary, and, whenever possible, delayed until confounding factors have been adequately addressed. Clear institutional protocols and ethical frameworks may help to ensure consistency and transparency in these decisions [162,163,164].

#### 3.9.4. Resource Allocation and Opportunity Cost

ECPR in inherently resource-intensive, requiring specialized personnel, advanced equipment and prolonged intensive care support. Its implementation may therefore impact the availability of critical care resource for other patients. From a system perspective, the allocation of resources to ECPR must be balanced against competing demands. This is particularly relevant in settings with limited ICU capacity or constrained healthcare budgets [157]. The regionalization of ECPR services may improve efficiency and outcomes but may also introduce disparities in access. Ensuring equitable patient selection and avoiding preferential allocation based on non-clinical factors remain key ethical priorities. When multiple eligible candidates compete for limited ECMO availability, prioritization should rely on objective clinical factors associated with the probability of meaningful recovery, including witnessed arrest, the no-flow interval, the low-flow duration, the initial rhythm, the reversibility of etiology and the comorbidity burden, rather than non-clinical or subjective criteria.

#### 3.9.5. Informed Consent and Family Communication

ECPR is typically initiated in emergency circumstances without the possibility of obtaining informed consent. This places additional responsibility on clinicians to engage in transparent and compassionate communication with families once the patient is stabilized. Families should be informed about the severity of the condition, the uncertainty of the outcome, the potential for neurological impairment, and the possible need for prolonged-life sustaining therapies. Ongoing communication is essential, particularly as prognostic clarity evolves [165,166].

## 4. Discussion

ECPR represents one of the most profound conceptual shifts in modern resuscitation medicine. Rather than attempting to restore spontaneous circulation through progressively ineffective compressive strategies, ECPR enables the immediate replacement of circulatory function, transforming cardiac arrest into a state of controlled extracorporeal perfusion [3,167]. However, the translation of this physiological advantage into consistent clinical benefit remains complex and highly context-dependent.

The available evidence suggests that ECPR is most effective when implemented within highly organized system capable of minimizing low-flow duration and rapidly providing definitive therapy whereas delayed or unstructured implementation may attenuate its benefit [11,12,13,61]. Another key aspect highlighted in this review is the central role of etiological diagnosis. ECPR should be viewed as a temporizing strategy that enables definitive treatment. The identification of reversible causes, such as acute coronary occlusion, pulmonary embolism or mechanical complications, is essential to convert extracorporeal stabilization into meaningful recovery [61,62,63,64,65]. Importantly, the presence of ECMO support modifies the therapeutic landscape, allowing for more controlled and targeted interventions compared to the pre-extracorporeal phase. At the same time, it exposes the risk of prolonged support in patients without reversible pathology. The physiological benefits of ECPR are counterbalanced by significant complications requiring advanced post-resuscitation management [33,85,86,93,94]. These complications are not merely technical issues but may directly influence survival and neurological outcome [168,169]. Effective post-resuscitation management therefore requires advanced critical care expertise, including neuroprotection, hemodynamic optimization and multiorgan support. From a broader perspective, ECPR challenges the traditional boundaries between resuscitation and intensive care. The transition from cardiac arrest to extracorporeal support creates a phase of therapeutic uncertainty in which prognosis is initially unclear and decision-making must evolve over time. This has important ethical implications, particularly regarding initiation in relation to borderline candidates, continuation in the absence of recovery and the allocation of limited healthcare resources. Overall, ECPR should be regarded as a highly selective and system-dependent intervention whose success relies on rapid deployment, appropriate patient selection, definitive therapy and advanced post-resuscitation care.

## 5. Conclusions

ECPR represents a paradigm shift from conventional, compression-based resuscitation toward system-based extracorporeal support. By restoring effective circulation, ECPR creates a therapeutic window for the diagnosis and treatment of reversible causes of cardiac arrest. However, its clinical benefit is not universal. Outcomes are critically dependent on patient selection, the timing of the initiation and the presence of structured systems of care capable of delivering rapid cannulation, etiological diagnosis and advanced post-resuscitation management. ECPR should therefore be regarded not as a standalone intervention, but as a component of an integrated resuscitation strategy. Its appropriate use requires the careful balancing of physiological potential, clinical indication and ethical considerations.

## 6. Future Directions

Future research should focus on refining patient selection, reducing variability in implementation and improving neurological prognostication. Advances in mobile ECPR programs, ventricular unloading strategies, extracorporeal technology and biocompatible circuit design may further improve outcomes while reducing complications. The development of sustainable and ethically balanced organizational models will also be essential to ensure equitable access and long-term feasibility.

## Figures and Tables

**Table 1:** Randomized controlled trials evaluating ECPR in refractory cardiac arrest. Outcomes across trials appear closely associated with system performance and low-flow duration rather than ECPR use alone. ECPR: extracorporeal cardiopulmonary resuscitation; CPR: cardiopulmonary resuscitation; ACLS: advanced cardiac life support; OHCA: out-of-hospital cardiac arrest; VF: ventricular fibrillation; VT: ventricular tachycardia; ROSC: return of spontaneous circulation; and PCI: percutaneous coronary intervention.

Trial	Population	Inclusion Criteria	Intervention	Primary Outcome	Key Results	System Characteristics/Low-Flow Duration	Limitations
ARREST 2020 [11]	OHCA, shockable rhythm	Refractory VF/VT, witnessed arrest, no ROSC	ECPR vs. standard ACLS	Survival to hospital discharge with favorable neurological outcome	Significantly higher survival in ECPR group; trial stopped early for benefit	Highly integrated system, rapid cath-lab cannulation, median low-flow duration ~59 min	Single-center, small sample size, early termination
Prague OHCA 2022 [12]	OHCA, shockable and non-shockable rhythm	Refractory cardiac arrest	Invasive strategy (ECPR +early transport +PCI) vs. standard care	180-day survival with good neurological outcome	No significant difference in primary outcome; signal of benefit in selected subgroups	Variable system performance, transport-related delays, median low-flow duration ~61 min	System heterogeneity, delayed cannulation in some patients
INCEPTION 2023 [13]	OHCA, shockable rhythm	Refractory cardiac arrest	ECPR vs. conventional CPR	30-day survival with favorable neurological outcome	No significant difference between groups	Multicenter implementation, median low-flow duration ~74 min, variable expertise	Multicenter variability, delayed cannulation, system inefficiency

**Table 2:** Clinical factor associated with favorable and unfavorable outcomes in ECPR. These factors should be interpreted within a clinical context and do not represent absolute inclusion or exclusion criteria. CPR: cardiopulmonary resuscitation.

Clinical Variable	Favorable Profile	Unfavorable Factors	Relative Prognostic Relevance
Witnessed arrest	Yes	No	High
Immediate bystander CPR	Immediate CPR/no-flow < 5 min	Prolonged no-flow interval	Very high
Initial rhythm	Shockable rhythm	Non shockable rhythm	High
Low-flow duration	<60 min	Prolonged low-flow duration	Very high
Age	Younger age (<65–70 years, relative criterion)	Advanced age with frailty/comorbidity	Moderate
Etiology	Presumed reversible etiology	Non-reversible or unknown etiology	High
Comorbidities	Limited comorbidity burden	Severe pre-existing disease	Moderate

**Table 3:** ECPR as a bridge-to-therapy: etiological diagnosis and definitive management pathways. CT: computed tomography; PCI: percutaneous coronary intervention; ECG: electrocardiogram; and ICD: implantable cardioverter-defibrillator.

Etiology	Diagnostic Tool	Definitive Treatment	Role of ECMO
Acute myocardial infarction	Coronary angiography	Percutaneous coronary intervention (PCI)	Bridge to revascularization
Pulmonary embolism	Echocardiography/CT	Surgical embolectomy or catheter-directed therapy	Bridge to reperfusion
Aortic dissection	CT/Echocardiography	Surgical repair	Bridge to surgery (or futility in non-candidates)
Primary arrhythmia	ECG	Antiarrhythmic therapy/ICD	Bridge to stabilization
Toxicological/metabolic causes	Laboratory test	Antidotes/targeted correction	Bridge to clearance and recovery

**Table 4:** Major complications of ECPR and targeted management strategies. IABP: intra-aortic balloon pump; CPR: cardiopulmonary resuscitation.

Complication	Mechanism	Clinical Impact	Management Strategy
Left ventricular distension	Increased afterload due to retrograde ECMO flow	Pulmonary edema; impaired myocardial recovery	Ventricular unloading (inotropes, IABP, Impella, and venting)
Differential hypoxia	Competition between native and ECMO flow (mixing point shift)	Cerebral and coronary hypoxia	Ventilatory optimization, ECMO flow adjustment, configuration change
Bleeding	CPR-related trauma, anticoagulation coagulopathy	Hemorrhage; increased mortality	Individualized anticoagulation, correction of coagulopathy
Limb ischemia	Femoral arterial cannulation	Tissue ischemia; potential limb loss	Distal perfusion cannula, vascular monitoring, early intervention

**Table 5:** Unloading/venting strategies during peripheral V-A ECMO. LV: left ventricle; IABP: intra-aortic ballon pump; and LA: left atrium.

Strategy	Mechanism	Advantages	Limitations
Inotropes and ECMO flow adjustment	Promote aortic valve opening and native LV ejection	Rapid and immediately available	May increase myocardial oxygen consumption and are often insufficient alone
Intra-aortic balloon pump (IABP)	Afterload reduction and coronary perfusion support	Widely available and relatively less invasive	Limited unloading capacity
Impella	Active LV drainage and forward flow support	Effective LV decompression and reduction in pulmonary congestion	Hemolysis, vascular complications, cost
Atrial septostomy	Left atrial decompression	Useful when direct LV access is difficult	Requires specific expertise and may provide indirect unloading
Surgical LV/LA venting	Direct ventricular or atrial decompression	Powerful unloading strategy	Invasive and generally requires surgical access

**Table 6:** Main technological features of contemporary ECMO systems.

System Feature	Contemporary Approach	Potential Clinical Relevance
Pump technology	Centrifugal pumps	Lower hemolysis, compact design, improved flow stability
Oxygenator membrane	Polymethylpentene hollow fiber membrane	Improved gas exchange durability and lower plasma leakage
Circuit coating	Heparin-coated or biocompatible surfaces	Reduced thrombogenicity and inflammatory activation
Monitoring systems	Integrated flow, pressure and saturation sensors	Earlier detection of circuit dysfunction
Portability	Miniaturized transport-compatible systems	Facilitates mobile ECMO and ECPR networks

## Data Availability

Not applicable.

## Abbreviations

The following abbreviations are used in this manuscript:

ECPR	Extracorporeal cardiopulmonary resuscitation
V-A ECMO	Veno-arterial extracorporeal membrane oxygenation
CPR	Cardiopulmonary resuscitation
OHCA	Out-of-hospital cardiac arrest
IHCA	Intrahospital cardiac arrest
